# Editorial: How underappreciated autoinflammatory mechanisms (innate immunity) dominate disparate autoimmune disorders

**DOI:** 10.3389/fimmu.2025.1762297

**Published:** 2025-12-19

**Authors:** Qiao Zhou, Yue Yang, Dennis McGonagle

**Affiliations:** 1 Department of Rheumatology and Immunology, Sichuan Provincial People’s Hospital, University of Electronic Science and Technology of China, Chengdu, China; 2 Clinical Immunology Translational Medicine Key Laboratory of Sichuan Province, Sichuan Provincial People’s Hospital, University of Electronic Science and Technology of China, Chengdu, China; 3 Department of Rheumatology and Immunology, Peking University People’s Hospital, Peking, China; 4 Leeds Institute of Rheumatic and Musculoskeletal Medicine (LIRMM), University of Leeds, Leeds, United Kingdom; 5 National Institute for Health Research (NIHR) Leeds Biomedical Research Centre (BRC), Leeds Teaching Hospitals, Leeds, United Kingdom

**Keywords:** IL-23 axis, NETs, neutrophil-fibroblast signaling, PAD activation, PRR checkpoints

The aim of this Research Topic was to investigate how innate immune pathways—often relegated to the periphery of “classical autoimmunity”—in fact organize, initiate, or amplify inflammation across a range of conditions historically classified as autoimmune. The contributions gathered here collectively propose a spectrum-based perspective. This view blurs rigid boundaries between autoimmunity and autoinflammation, highlights neutrophil- and barrier-centric mechanisms, and suggests therapeutic strategies that either target upstream innate triggers or modulate adaptive immunity with an awareness of innate mechanisms. Together, they call for an integrated immune paradigm, one that better reflects the biology of human disease and offers more actionable insights for clinical practice.

At the conceptual core, Abacar et al. revisit the intellectual history from horror autotoxicus to “horror autoinflammaticus”, and then deliberately stitch these concepts back together into a unified continuum. They show how diseases long considered archetypes of adaptive autoimmunity (e.g., RA and SLE) frequently rely on innate events for their initiation. It also details how barrier failure and dysbiosis drive adaptive immune cascades in mucosal disorders. Their review further profiles the disease landscape from MHC-II-associated disorders to MHC-I-opathies and the bridging roles of unconventional lymphocytes. The argument is made that numerous “autoimmune” phenotypes are more accurately understood as composite, multi-stage processes where innate and adaptive immunity act sequentially or in concert.

Two original studies then ground this integrative view in empirical data. In rheumatoid arthritis, Li et al. dissect the synovial microenvironment and elevate neutrophils—often overlooked in the discussions of RA pathogenesis—at a center role(2). Using single-cell and bulk transcriptomic integration with machine-learning prioritization, they identify seven major cellular compartments, with neutrophils being the most abundant. They map extensive crosstalk between neutrophils and fibroblasts (including MIF, collagen, and Fn1 signaling) and develop a clinically relevant, four-gene diagnostic model related to NETs (CRYBG1, RRM2, MMP1, SLC19A2). Their work reframes RA as an ecosystem where neutrophil extracellular traps (NETs) contribute to fibroblast pathogenicity and shape T-cell antigen presentation, supporting the idea that “autoimmune RA” is nevertheless scaffolded by neutrophil-driven innate events.

Those RA insights resonate with the continuum articulated by Abacar et al., who describe how citrullination and PAD-driven antigen editing—processes classically invoked to explain ACPA-positive adaptive autoimmunity—are themselves initiated by innate signals such as macrophage activation, Ca^2+^ flux, and NETosis. This sequence—innate ignition, adaptive specification, innate effector return—helps explain why therapies targeting both sides of immunity can be effective in the same patient and even at different disease stages.

On the mucosal end of the spectrum, Abacar et al. also emphasize how barrier disruption, PRR sensing, and innate gene variants (e.g., NOD2, CARD9) establish a foundation for the IL-23/Th17 axis and subsequent adaptive immune circuitry in IBD. This again illustrates disease trajectories that start as autoinflammation and progressively acquire adaptive hallmarks.

The therapeutic implications of an integrated view are brought into focus by Liu et al., who apply a Bayesian network meta-regression to biologics in lupus nephritis and, crucially, adjust for follow-up duration. They confirm that several agents—particularly obinutuzumab—can achive faster remission early on. This reveals a clinically meaningful “time-window” of advantage versus standard therapy alone. As follow-up extends, these relative differences attenuate, underscoring standard care as a durable base while clarifying when targeted biologics provide the most added value. Despite the multifaceted involvement of innate immunity in SLE, B-cell depletion and thus suppression of central adaptive immunity responses thus remains central to SLE therapeutics. This time-sensitive perspective argues for treatment strategies tailored to disease stage, acknowledging the shifting balance between innate and adaptive immune dominance across the course of LN.

Finally, Chen et al. review mesenchymal stem cells (MSCs) as potential immunologic “equalizers”, capable of fine-tuning both innate and adaptive immunity via direct contact and paracrine programs, including EVs. Mechanistically, the review details how MSCs can promote regulatory T-cell circuits (e.g., via PD-L1/PD-1) and reprogram innate effector cells (e.g., macrophages, dendritic cells, NK cells). This positions MSC-based therapy as a platform capable of dampening hyperreactive immune loops, irrespective of whether they originate from innate or adaptive triggers. In conditions across the autoinflammatory-autoimmune spectrum, such dual modulation could be particularly attractive when the initial trigger is innate but the clinical phenotype has matured into mixed, relapsing immunopathology.

Cross-cutting themes and future directions

Three practical messages emerge. First, timing is critical. Innate immune circuits can dominate early phases and be most amenable to intervetion then (e.g., the early advantage of biologics in LN), while adaptive immune plateaus may govern long-term maintenance—advocating for adaptive trial designs and dynamic treatment algorithms aligned with disease phase. Second, neutrophils are key players. Across different tissues, NETs and neutrophil-stromal interactions appear capable of initiating site-specific pathology and antigen presentation. This makes neutrophil biology a rich source for novel biomarkers and therapeutic targets (e.g., diagnostic models, NET-directed strategies, MIF pathway inhibition). Third, bridging therapies hold promise. Therapeutic platforms like MSCs, and, more broadly, strategies that temper inappropriate innate activation while preserving essential host defense, may complement antigen-specific or B/T-cell-directed agents, especially in diseases with mixed-phenotype.

In summary, this Research Topic reframes disparate autoimmune diagnoses as variations on a shared choreography in which innate immunity is not merely the opening act but often the choreographer—priming tissues, instructing stromal partners, and shaping the subsequent adaptive repertoire. The articles collected here delineate key mechanistic threads (NETs, PAD activation, PRR checkpoints, IL-23 axis, neutrophil-fibroblast signaling) and provide methodological and therapeutic frameworks (time-aware evidence synthesis; MSC-based modulation; biomarker-guided models) to translate this refined understanding into clinical practice. We anticipate that future work combining longitudinal immune phenotyping with precise interventional timing, and pairing innate-targeted agents with adaptive immune controllers, will lead to more durable disease control with fewer trade-offs—a direction firmly rooted in the integrative biology emphasized throughout these contributions.

